# Activating transcription factor 5 (ATF5) promotes tumorigenic capability and activates the Wnt/b-catenin pathway in bladder cancer

**DOI:** 10.1186/s12935-021-02315-x

**Published:** 2021-12-11

**Authors:** Junhao Zhou, Hu Tian, Xi Zhi, Zhuoyu Xiao, Taoyi Chen, Haoyu Yuan, Qi Chen, Mingkun Chen, Jiankun Yang, Qizhao Zhou, Kangyi Xue, Wenbin Guo, Ming Xia, Jiming Bao, Cheng Yang, Haifeng Duan, Hongyi Wang, Zhipeng Huang, Ting Zhu, Cundong Liu

**Affiliations:** 1 Department of Urology, The Third Affiliated Hospital of Southern Medical University, Guangzhou, 510630 Guangdong People’s Republic of China; 2 Department of Urology, The First People’s Hospital of Zhaoqing, Zhaoqing, 526020 China; 3 Department of Laboratory Medicine, Affiliated Cancer Hospital & Institute of Guangzhou Medical University, Guangzhou, 510095 Guangdong People’s Republic of China

**Keywords:** ATF5, Tumorigenicity, Recurrence, Wnt/β-catenin signaling, Bladder cancer

## Abstract

**Background:**

In bladder cancer, up to 70% of patients will relapse after resection within 5 years, in which the mechanism underlying the recurrence remains largely unclear.

**Methods:**

Quantitative real-time PCR, western blot and immunohistochemistry were conducted. The assays of tumor sphere formation and tumor xenograft were further performed to assess the potential biological roles of ATF5 (activating transcription factor 5). Chromatin immunoprecipitation-qPCR and luciferase activity assays were carried out to explore the potential molecular mechanism. A two-tailed paired Student's *t*-test, χ^2^ test, Kaplan Meier and Cox regression analyses, and Spearman's rank correlation coefficients were used for statistical analyses.

**Results:**

ATF5 is elevated in bladder urothelial cancer (BLCA) tissues, especially in recurrent BLCA, which confers a poor prognosis. Overexpressing ATF5 significantly enhanced, whereas silencing ATF5 inhibited, the capability of tumor sphere formation in bladder cancer cells. Mechanically, ATF5 could directly bind to and stimulate the promoter of *DVL1* gene, resulting in activation of Wnt/β-catenin pathway.

**Conclusions:**

This study provides a novel insight into a portion of the mechanism underlying high recurrence potential of BLCA, presenting ATF5 as a prognostic factor or potential therapeutic target for preventing recurrence in BLCA.

## Background

Globally, bladder cancer is the 10th most prevalent cancer type, with about 549,000 newly diagnosed patients in 2018 [1]. Unfortunately, up to 70% of these patients will relapse upon transurethral resection of bladder cancer [2], which greatly increases the suffering of patients.

Cancer recurrence is highly associated with cancer cell drug resistance and high tumorigenic capability [3]. Moreover, these characteristics could be examined in a small cell subpopulation in bladder cancer tissue, which are called the cancer stem cells (CSC) or the tumor initiating cells (TICs) [3–5]. TICs, with high self-renewal potential, are considered to contribute to tumor heterogeneity [6, 7]. More and more researches support the viewpoint that TICs participate in tumorigenesis and regional recurrence in bladder cancer subgroups [4, 8, 9]. Accordingly, targeting these TIC-like features may be an available therapy for decreasing tumor recurrence.

Ectopic stimulation of the Wnt/β-catenin pathway is required for drug-resistance and self renewal in colorectal TICs, which confers a poor prognosis [10, 11]. In bladder cancer, the Wnt/β-catenin pathway takes part in epithelium stem cell maintenance [12], indicating that changes in the vital pathway may lead to the tumorigenic potential of bladder cancer. Furthermore, genetic variants in this stem cell pathway are proven to modulate the etiology of bladder cancer [13]. Therefore, investigation on the mechanisms underpinning the ectopic stimulation of Wnt/β-catenin pathway in bladder cancer may provide valuable therapy target for preventing recurrence.

Activating transcription factor 5 (ATF5) belongs to the basic leucine zipper family of transcription factors. ATF5 participates in transcriptional responses of multiple cellular stressors, such as cytosolic heat shock response [14], endoplasmic reticulum unfolded protein response [15]. Several studies have reported high expression of ATF5 in undifferentiated neural progenitor cells/stem cells [16–19]. It plays a vital role in regulating cellular differentiation in various tissues, including the brain [20], liver [21], fat [22], and bone [23]. These functions that regulate the growth and differentiation in progenitor/stem cells indicate the potential role of ATF5 in the self-renewal and differentiation of TICs. Furthermore, the molecular mechanism underpinning the oncogenic role of ATF5 is largely unclear.

Herein, ATF5 was detected to be significantly upregulated in bladder urothelial carcinoma (BLCA) tissues, especially in recurrent BLCA. Ectopic ATF5 was significantly related to the clinicopathological characteristics and relapse-free survival rate of BLCA patients. Over-expressing ATF5 enhanced, whereas silencing ATF5 decreased, tumorigenic capability of BLCA in vitro and in vivo. Mechanistically, ATF5 transcriptionally upregulated *DVL1* expression by binding its promoter region. This study suggests that ATF5 plays a key role in tumorigenic capability of BLCA, and might be a valuable prognosis marker and potential therapeutic target for BLCA patients.

## Materials and methods

### Cell culture

Bladder cancer cells SW780 and UM-UC-3 were bought from the American Type Culture Collection (ATCC, USA) and grown in Dulbecco’s modified Eagle medium (Invitrogen, Carlsbad, California, USA) with fetal bovine serum (10%; HyClone, Logan, Utah, USA). Human lymphatic endothelial cells (HLECs) were acquired from ScienCell Research Laboratories (Carlsbad, California, USA) and maintained based on the manufacturer’s directions. Cell incubation was done at 37 °C in a 95% air and carbon dioxide (5%) environment.

### Patients and collections of tissue samples

In this study, 140 primary BLCA tissues that had been embedded in paraffin were used. Diagnosis had been histopathologically performed at The Third Affiliated Hospital of Southern Medical University and Affiliated Cancer Hospital & Institute of Guangzhou Medical University from 2010 to 2015. Clinical as well as clinic-pathological classification and staging was evaluated based on the American Joint Committee on Cancer criteria. Clinically relevant information for the 140 study participants was listed in Table 1. Additionally, four fresh non-relapse BLCA specimens and four relapse BLCA specimens were acquired. The Clinical Trials Ethics Committee (The Third Affiliated Hospital of Southern Medical University, and Affiliated Cancer Hospital & Institute of Guangzhou Medical University) approved this research.

**Table 1:** Clinicopathological characteristics of clinical samples and expression of ATF5 in 140 bladder cancer

Characteristics	No. patients	(%)
Age (years)		
≤ 60	62	(44.3)
> 60	78	(55.7)
Gender		
Male	106	(75.7)
Female	34	(24.3)
Grade		
Low grade	103	(73.6)
High grade	37	(26.4)
Stage		
Ta, T1	110	(78.6)
T2–T4	30	(21.4)
Tumor size		
≤ 3 cm	105	(75.0)
> 3 cm	35	(25.0)
Multiplicity		
1	97	(69.3)
> 1	43	(30.7)
Expression of ATF5		
High expression	65	(46.4)
Low expression	75	(53.6)
Recurrence		
Yes	38	(27.1)
No	102	(72.9)
Progression		
Yes	26	(18.6)
No	114	(81.4)

### RNA extraction and quantitative real-time PCR (qRT-PCR)

Isolation of total RNA from cells was done using the TRIzol reagent (Invitrogen, Cat No. 15596018) as instructed by the manufacturer and used for cDNA synthesis with random primers. Normalize the expression data to *GAPDH* gene. Expressions were calculated using the 2^−[(Ct of *gene*)−(Ct of *GAPDH*)]^ method, whereby Ct denotes each transcripts’ threshold cycles.

### Western blot (WB) assay

Western blot assay was conducted as reported previously [24]. The antibodies used were: anti-ATF5 (Cat. No.HP001912, Sigma, St. Louis, MO, USA), anti-active-β-catenin (Cat. No.05-665-25UG, Pharmingen/BD Biosciences, Bedford, MA, USA), anti-DVL1 (Cat. No.ab106844, Abcam, Cambridge, MA, USA), anti-ABCG2 (Abcam, Cat. No. ab108312), anti-SOX2 (Abcam, Cat. No.ab92494) and anti-GAPDH antibody (Cat. No.T6199, Sigma-Aldrich).

### Immunohistochemistry (IHC)

IHC was carried out in 140 BLCA tissues, which were detected with an anti-ATF5 antibody (Cat. No.HP001912, Sigma-Aldrich) as previously reported [24]. Immunostaining degree, separately scored by two pathologists working independently and who had been blinded to histopathological features as well as patient information, was evaluated by staining indices (SI). The SI was determined as the product of staining intensity score and tumor grade cell proportions. Grading of tumor cell proportions was as: 0, absent positive tumor cells (PTC); 1, < 2% PTC; 2, 2–8% PTC; 3, 8–20% PTC; and 4, > 20% PTC. Assessment of staining intensities was as: 1, absent staining; 2, weakly (light yellow) stained; 3, moderately (yellow–brown) stained; and 4, strongly (brown) stained. Protein expression evaluated by SI had possible scores of 0, 1, 2, 3, 4, 6, 8, 9, 12 or 16. The sample with SI ≥ 8 was defined as highly expressed, while the sample with SI < 8 was defined as low expressed. Determination of the cut-off value was done according to the heterogeneity measure using the log rank test in accordance with overall survival.

### Plasmids, retroviral infection, and transfection

Amplification of the human *ATF5* gene from cDNA was done by PCR and later cloned into a pcDNA4 lentiviral vector. For silencing ATF5, cloning of 2 human ATF5-targeting short hairpins RNA (shRNA) sequences into a PLKO.1 (OligoEngine, Washington, USA) was done to enable the generation of corresponding pSUPER. retro. ATF5-RNAi(s). SW780 or UM-UC-3 cells were seeded in culture plates (2 × 10^6^ cells/p100 plate). Transfection was done using 10 ug of designated plasmids. Cells stably expressing ATF5 or ATF5 shRNA were obtained through retroviral infection of the 293FT cells. These cells were treated for 3 days using puromycin (3.33 µg/mL). The promoter region of the human *DVL1* gene, which had been generated via PCR-amplification from SW780 cells were cloned into NheI/BglII sites of the pGL3-basic luciferase reporter plasmids (Promega,Madison, WI, USA) in order to establish DVL1 luciferase reporters. Tables 2 and 3 shows the primers used in this study.

**Table 2:** Primers used for gene detection

Gene	Sequence (5′-3′)
*ATF5*	F: TGGCTCGTAGACTATGGGAAA
	R: ATCAACTCGCTCAGTCATCCA
*GAPDH*	F: TGTGGGCATCAATGGATTTGG
	R: ACACCATGTATTCCGGGTCAAT
*DVL1*	F: GAGGGTGCTCACTCGGATG
	R: GTGCCTGTCTCGTTGTCCA
*NANOG*	F: TCCCGAGAAAAGATTAGTCAGCA
	R: AGTGGGGCACCTGTTTAACTT
*SOX2*	F: CTCGTGCAGTTCTACTCGTCG
	R: AGCTCTCGGTCAGGTCCTTT
*ABCG2*	F: CAGGTGGAGGCAAATCTTCGT
	R: ACCCTGTTAATCCGTTCGTTTT
*TCF1*	F: CAGAGGAGAGGAACCAAGCTA
	R: GCAACTCGGGACATAAAGCC
*CCND1*	F: GCTGCGAAGTGGAAACCATC
	R: CCTCCTTCTGCACACATTTGAA
*CD44*	F: CTGCCGCTTTGCAGGTGTA
	R: CATTGTGGGCAAGGTGCTATT
*JUN*	F: CATTGTGGGCAAGGTGCTATT
	R: ACAGAGCGAGTGAAAATGTGTAT

**Table 3:** The siRNA targets sequence of *DVL1*

Gene	Sequence (5′-3′)
*siDVL1#1*	sense: CCAAGAUUAUCUACCACAUTT
	antisense: AUGUGGUAGAUAAUCUUGGTT
*siDVL1#2*	sense: CCAAGCUUCCCUGCUUCAATT
	antisense: UUGAAGCAGGGAAGCUUGGTT
*siDVL1#3*	sense: GCAUCUACAUUGGCUCCAUTT
	antisense: AUGGAGCCAAUGUAGAUGCTT

### Tumor sphere formation test

Cells (5 × 10^2^) were plated in 6-well ultralow cluster plates and incubated for 10 to 12 days. Incubation of tumor spheres was done in serum free DMEM/F12 (Invitrogen, Cat. No.88215) with epidermal growth factor (EGF, 20 ng/mL, Cat. No.37000015, PeproTech, Rocky Hill, USA), B27 (2%, Cat. No.12587010, Invitrogen), basic fibroblast growth factor (20 ng/mL, Cat. No.100-18B, bFGF, PeproTech), insulin (5ug/mL, Cat. No.100-11, PeproTech), and BSA (0.4%, Cat. No.A1933-1G, Sigma-Aldrich). After 10–12 days, tumor spheres (spherical, tight, non-adherent masses that were larger than 50 µm in diameter) were counted and imaged by inverse microscopy. The efficiency of sphere formation was calculated using the formulacolonies/input cells × 100%.

### Luciferase reporter test

Luciferase reporter test was conducted as described previously. In short, in triplicates, bladder cancer cells (3 × 10^4^ cells per well) were plated in 24-well plates and incubated for 24 h. Transfection of specified plasmids as well as a pRL-TK Renilla plasmid (1.5 ng) was done using the Lipofectamine 3000 Reagent (Cat. No.L3000008, Thermo Fisher Scientific). Then, 48 h after transfection, the luciferase as well as Renilla signals were assessed by the Dual Luciferase Reporter Assay Kit (Cat. No.E1980, Promega) as instructed by the manufacturer.

### Chromatin immunoprecipitation-qPCR (ChIP-qPCR)

The ChIP assay was carried out as previously reported [24]. In short, crosslinking was carried out using formalin (1%) followed by cell lysis in sodium dodecyl sulfate (SDS) buffer. Then DNA fragmentation was achieved by sonication. ChIP for ATF5 was carried out with a Flag antibody (Sigma, SAB4301135). Fragments of DNA that had been eluted were detected by qPCR. The primer used for ChIP assay was shown as follow: *DVL1*primer 5:5′-TTGGAATGAGGCACAGGG-3′, 5'-GACAGAAAACTGCCCACC-3′.

### Tumor xenograft

Male BALB/c nude mice (5–6-weekold, 16–18 g) were bought from Guangdong Experimental Animal Center (Guangdong, China) and housed in an animal holding facility on a 12 h dark/light cycle. Randomly, mice were allocated in to four groups (n = 6 per group). Inguinal folds of mice were inoculated with SW780 cells (1 × 10^6^ cells), which had been stably transfected with RNAi-vector, ATF5-RNAi#1, ATF5 or vector, with Matrigel (to a final concentration of25%). An external caliper was used to assess tumor volumes, which were determined by the equation (L × W2)/2. Then, 36 d post-inoculation, mice were euthanized by quick intraperitoneal injection of 100 μg/g pentobarbital sodium, and sacrificed for tumor excision.

### Bioinformatics analysis

The BLCA database from The Cancer Genome Atlas (TCGA) was analyzed by Gene set enrichment analysis (GSEA, http://software.broadinstitute.org/gsea/msigdb/

index.jsp) software program (version 2.2.3).

### Statistical analysis

The SPSS v.21.0 software (SPSS Inc., New York, NY, USA) was used for all analyses. Comparison of means between groups was done by a two-tailed paired Student’s *t*-test. The association between ATF5 levels and clinic-pathological characteristics was calculated by χ^2^ test. Kaplan Meier was used for establishment of survival curves, which were compared by log-rank test. Then, survival data were calculated by univariate as well as multivariate Cox regression analyses. The bivariate correlation between variables was evaluated by Spearman's rank correlation coefficients. The *P-*value < 0.05 was the threshold for significance.

## Results

### Ectopic ATF5 expression is correlated with bladder cancer recurrence

**Fig. 1:**
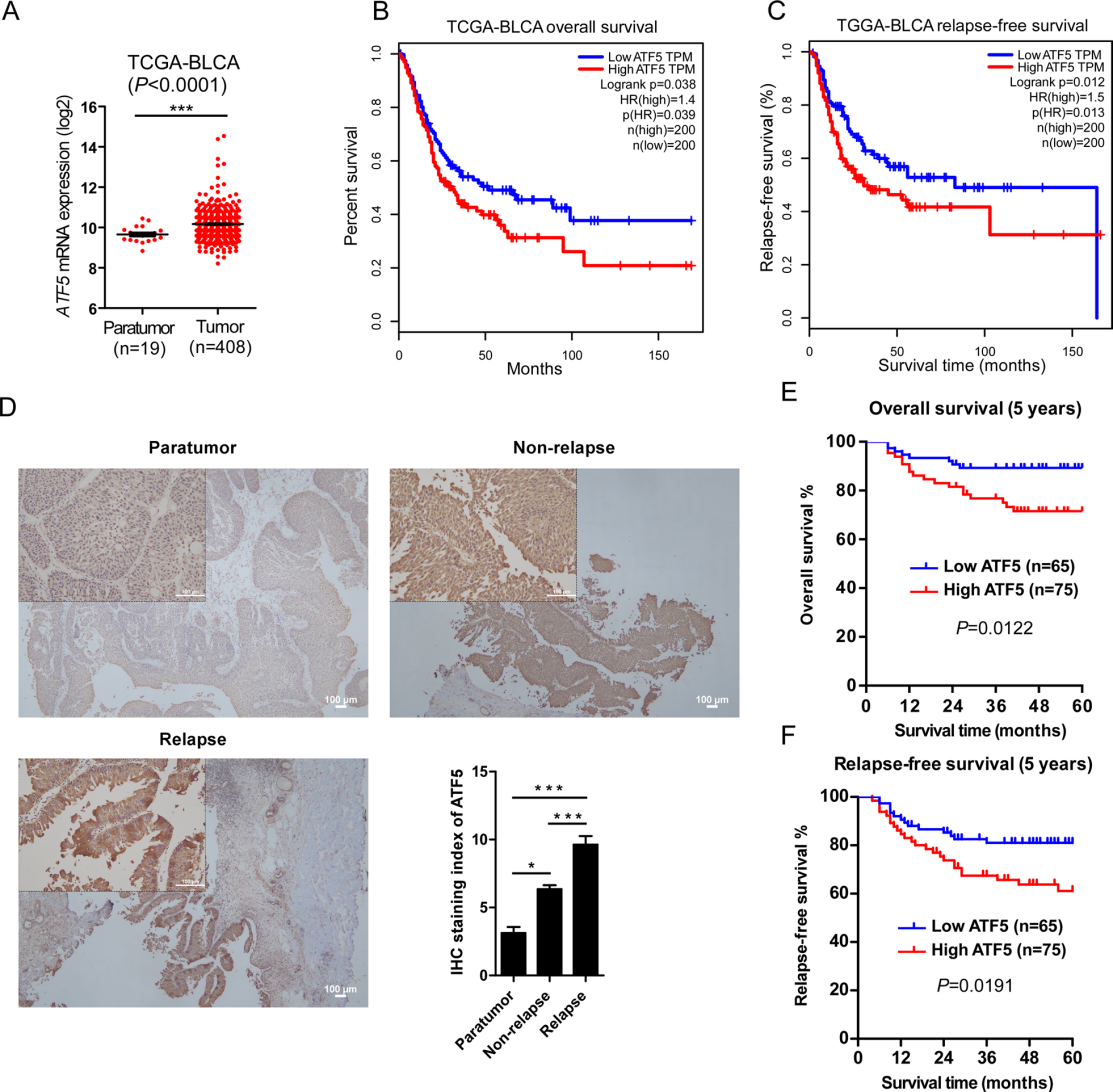
Ectopic ATF5 expression is correlated with recurrence in bladder cancer. **A** *ATF5* mRNA levels in the TCGA-BLCA dataset. **B** Overall survival of patients with low versus high *ATF5* gene expression in TCGA-BLCA dataset. **C** Relapse-free survival of patients with low versus high *ATF5* gene expression in TCGA-BLCA dataset. **D** Representative images of IHC staining in 102 non-relapse BLCA tissues and 38 BLCA tissues that recurred after therapy (left). Statistic analyses of the average staining index of ATF5 staining in paratumor bladder tissues and BLCA tissues (right). **E** Overall survival of BLCA patients with low versus high ATF5 expression. **F** Relapse-free survival of BLCA patients with low versus high ATF5 expression. Bars represent the mean ± SD of three independent experiments; *P < 0.05, **P < 0.01, ***P < 0.001. ATF5, activating transcription factor 5; TCGA, the cancer genome atlas; BLCA, bladder urothelial carcinoma; IHC, immunohistochemistry

To investigate the significance of ATF5 in BLCA as well as its potential oncogenic mechanism, *ATF5* mRNA expression was preliminary analyzed between BLCA tissues and paratumor tissues in an available BLCA data from The Cancer Genome Atlas (TCGA) database. Expression levels of the *ATF5* gene in BLCA were markedly elevated than those of adjacent normal bladder tissues (Fig. 1A). Its gene expression was negatively correlated with overall survival outcomes of BLCA patients (*P* = 0.039, Fig. 1B). Furthermore, there was a negative association between *ATF5* gene expression and relapse-free survival outcomes (*P* = 0.013, Fig. 1C), indicating that *ATF5* might be a predictor of recurrent risk in BLCA.

To assess the correlation of ATF5 expression and clinicopathological characteristics of BLCA, 140 BLCA specimens (102 non-relapse BLCA tissues and 38 recurrent BLCA tissues after treatment) were stained using a human ATF5 antibody. ATF5 expression in BLCA specimens was markedly elevated than in paratumor tissues (Fig. 1D). In addition, ATF5 expression levels in recurrent BLCA specimens were markedly elevated than those of non-relapse specimens (Fig. 1D). Statistic analysis of the 140 BLCA samples revealed that ATF5 expressions were associated with clinical grade (*P* = 0.034), stage (*P* = 0.014), tumor multiplicity (*P* = 0.004), recurrence (*P* = 0.022) as well as progression (*P* = 0.016), however, they were not correlated with gender, tumor size or age (Table 4). Importantly, Kaplan–Meier as well as log-rank analyses demonstrated that ATF5 expressions were negatively related to overall survival outcomes (*P* = 0.012, Fig. 1E) and relapse-free survival outcomes (*P* = 0.019, Fig. 1F). These results showed that ectopic ATF5 expression was closely related to recurrence, which predicted a poor prognosis of BLCA.

**Table 4:** Correlation between ATF5 expression and clinicopathologic charateristics of 140 bladder cancer

Characteristic	ATF5 protein level		Chi-square test
	High (65)	Low(75)	*P* value
Age (> 60 versus ≤ 60 years)	36/29	42/33	0.942
Gender (M versus F)	46/19	60/15	0.238
Grade (low versus high)	42/23	61/14	0.034*
Stage (Ta-1 versus T2-4)	45/20	65/10	0.014*
Tumor Size (> 3 cm versus ≤ 3 cm)	21/44	14/61	0.079
Multiplicity (single versus multiple)	37/28	60/15	0.004**
Recurrence (Yes versus No)	24/41	14/61	0.022*
Progression (Yes versus No)	18/47	8/67	0.016*

*P < 0.05, **P < 0.01

### Elevated ATF5 expression in bladder cancer cells enhanced a tumor initiating cells (TIC)-like phenotype

**Fig. 2:**
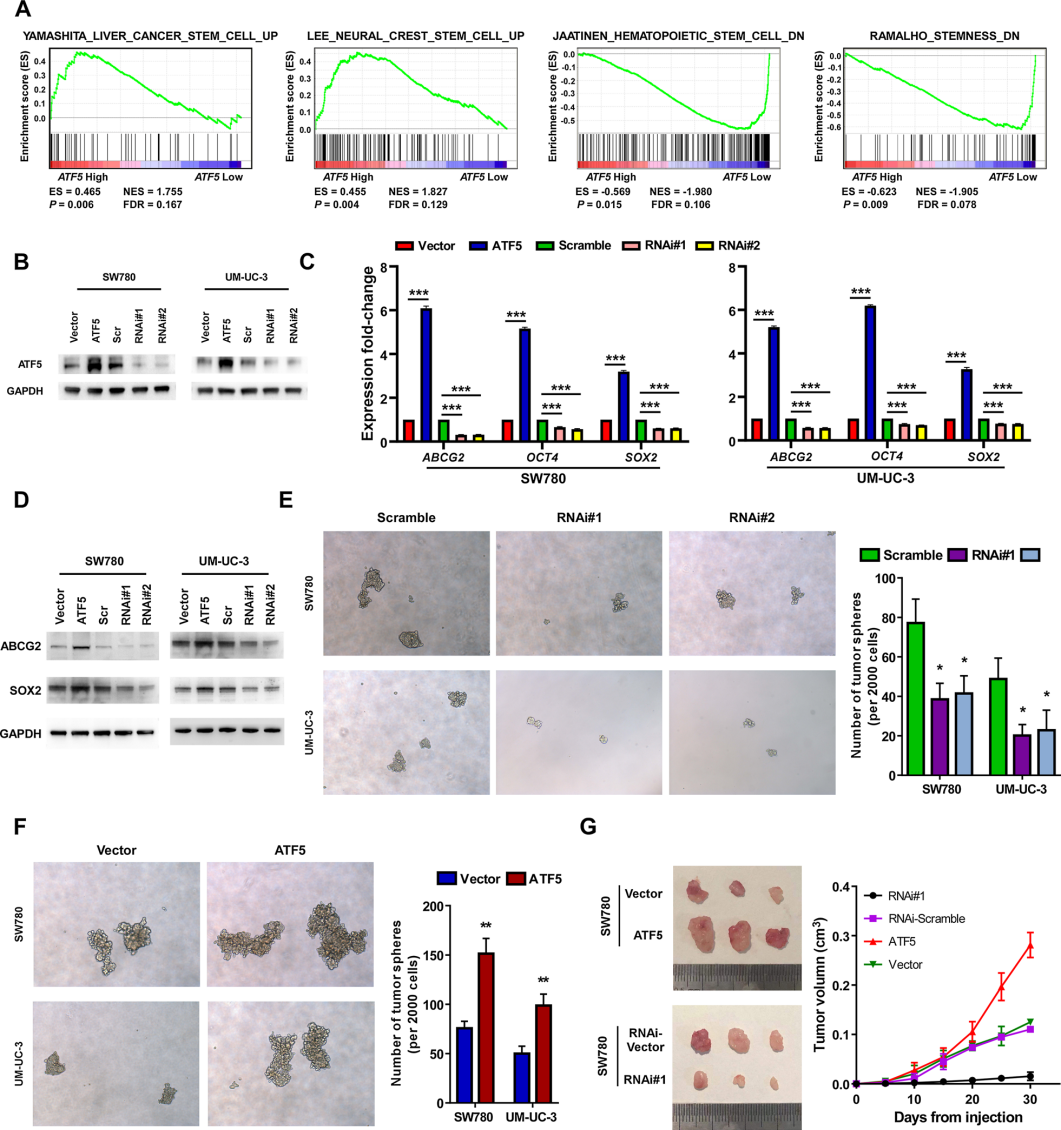
ATF5 upregulation in bladder cancer cells enhances a TIC-like phenotype. **A** GSEA of correlations between *ATF5* gene expression and stemness-related gene signatures (YAMASHITA_LIVER_CANCER_STEM_CELL_UP, LEE_NEURAL_CREST_STEM_CELL_UP, JAATINEN_HEMATOPOIETIC_STEM_CELL_DN, RAMALHO_STEMNESS_DN). **B** Western blotting detection of ATF5 in the indicated ATF5-upregulation, ATF5-knockdown, or control cells. GAPDH was used as the loading control. **C**, **D** qRT-PCR (**C**) and WB (**D**) detection of stemness-related markers (*ABCG2*, *OCT4*, *SOX2*) in overexpressing, downregulating ATF5 or control cells. **E**, **F** Representative images and quantitative analyses of tumor sphere formation in ATF5-upregulation cells (**E**, × 100), ATF5-knockdown cells (**F**, × 100) or control cells. **G** Tumors formed by ATF5-overexpressing bladder cancer cells were larger than control tumors. In contrast, tumors formed by ATF5-knockdown cells were smaller than those formed by RNAi-vector cells. Representative images of tumors were displayed (left). Tumor growth curves after injection of indicated cells (right). Bars represent the mean ± SD of three independent experiments; *P < 0.05, **P < 0.01, ***P < 0.001. GSEA, gene set enrichment analysis; TIC, tumor initiating cells; qRT-PCR, quantitative real-time polymerase chain reaction; WB, western blotting

GSEA analysis was conducted to further assess the potential biological roles of ATF5 in cancers. GSEA analysis of the public BLCA database from TCGA showed a positive correlation between ATF5 expression and stemness signatures (Fig. 2A), which was tightly associated with cancer recurrence. Moreover, qRT-PCR and WB showed that the expression levels of stemness-associated biomarkers, such as *OCT4*, *ABCG2* and *SOX2* were markedly elevated in ATF5-overexpressed SW780 and UM-UC-3 cells, whereas reduced in ATF5-silenced bladder cancer cells (Fig. 2B–D). Subsequently, the tumor sphere formation test was carried out to investigate the role of ATF5 in self renewal of spherogenic bladder cancer cells. ATF5-knockdown cells formed fewer spheres with fewer cells relative to the control group (Fig. 2E), whereas ATF5-overexpressing cells formed more spheres with more cells (Fig. 2F). These findings indicated that ATF5 upregulation enhanced tumorigenic capability of bladder cancer cells in vitro.

To evaluate the oncogenic significance of ATF5, through the subcutaneous route, bladder cancer cells were inoculated into the inguinal area of nude mice. Tumor formation in ATF5-overexpressing SW780 cells was markedly greater, relative to the control group (Fig. 2G). Besides, ATF5-knockdown cells formed significantly smaller tumors with lower tumorigenic capability (Fig. 2G). This data demonstrated that ATF5 upregulation enhanced bladder cancer cell tumorigenic capability in vivo.

### ATF5 upregulation stimulates Wnt/b-catenin signaling pathway

**Fig. 3:**
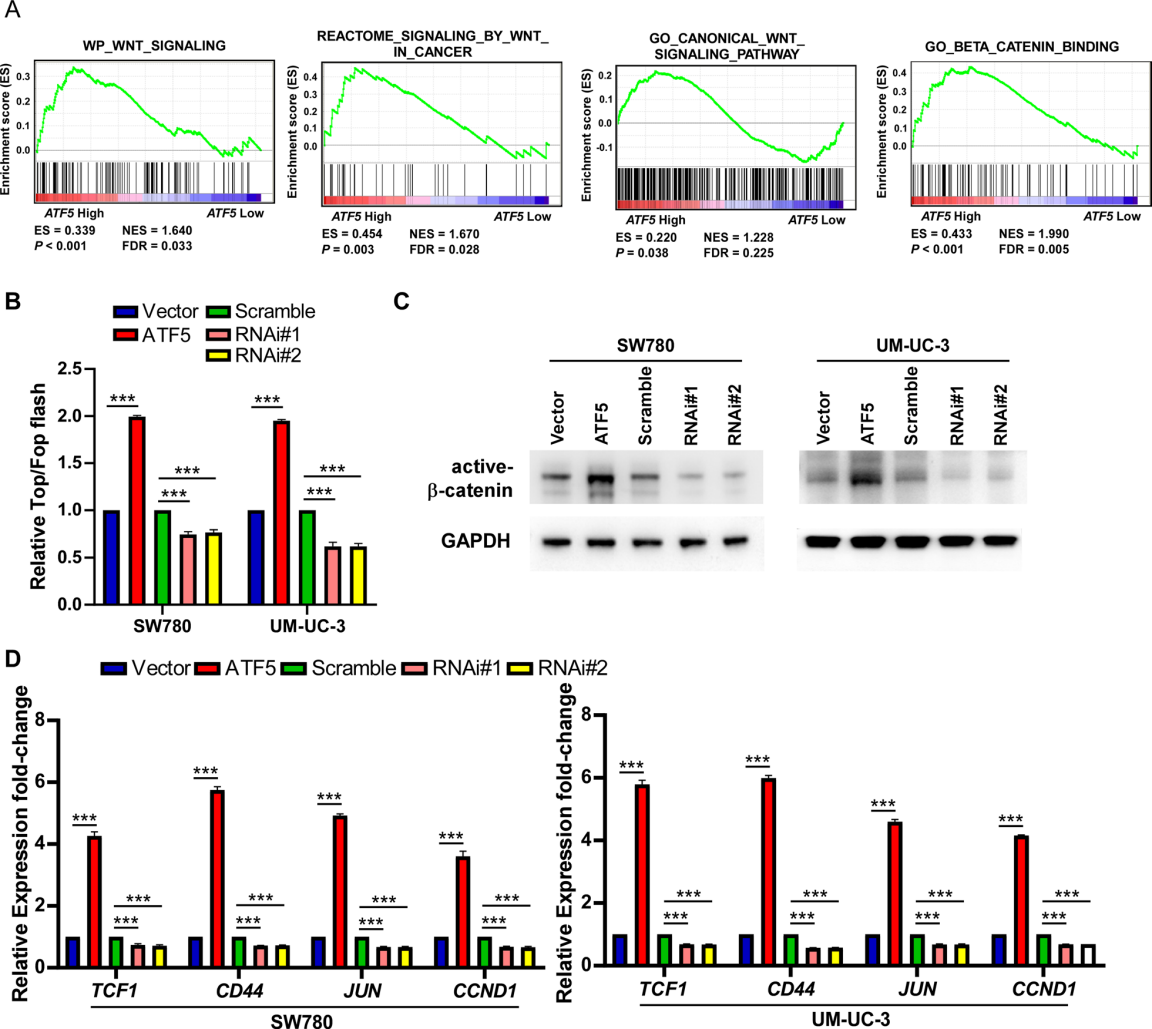
ATF5 upregulation stimulates Wnt/β-catenin pathway. **A** GSEA showing the positive correlations between *ATF5* gene expression and Wnt pathway from publicly available profiles (WP_WNT_SIGNALING, REACTOME_SIGNALING_BY_WNT_IN_CANCER, GO_CANONICAL_WNT_SIGNALING_PATHWAY, GO_BETA_CATENIN_BINDING). **B** Luciferase reporter test of TOP/FOP transcriptional activity in ATF5-upregulation and ATF5-knockdown SW780/UM-UC-3 cells compared with control cells, respectively. **C** WB detection of the active-β-catenin upon overexpression or knockdown of ATF5 in SW780 and UM-UC-3 cells relative to control groups. **D** qRT-PCR assay of genes expression of Wnt/β-catenin downstream targets (*TCF1*, *CD44*, *JUN*, *CCND1*,) in overexpressing, downregulating ATF5 or control cells. Bars indicate the mean ± SD of three independent experiments; *P < 0.05, **P < 0.01, ***P < 0.001. GSEA, gene set enrichment analysis; WB, western blotting; qRT-PCR, quantitative real-time polymerase chain reaction

To investigate the mechanism underpinning ATF5-mediated TIC-like phenotype in BLCA, GSEA of TCGA-BLCA datasets was performed to analyze the potential correlation of *ATF5* expressions and genes regulated by multiple signaling signatures. *ATF5* gene expression was positively related to Wnt-related and beta-catenin-related gene signatures (Fig. 3A), implying that ATF5 stimulated Wnt/β-catenin signaling. Moreover, TOP/FOP assay demonstrated that ATF5 upregulation significantly promoted, whereas ATF5 knockdown repressed the transcriptional stimulation of TCF/LEF in the SW780 as well as UM-UC-3 cells (Fig. 3B). Consistently, WB anslysis demonstrated that ATF5 upregulation significantly elevated the active-β-catenin signals, while ATF5 attenuation decreased them (Fig. 3C). Expressions of downstream genes of the Wnt/β-catenin signaling, such as *CD44*, *TCF1*, *JUN* and *CCND1*, were elevated in ATF5-overexpressing cells, whereas these targets were reduced in ATF5-knockdown cells (Fig. 3D). Overall, Elevated ATF5 could stimulate Wnt/β-catenin pathway.

### ATF5 binds, and activates the promoter of DVL1 gene

**Fig. 4:**
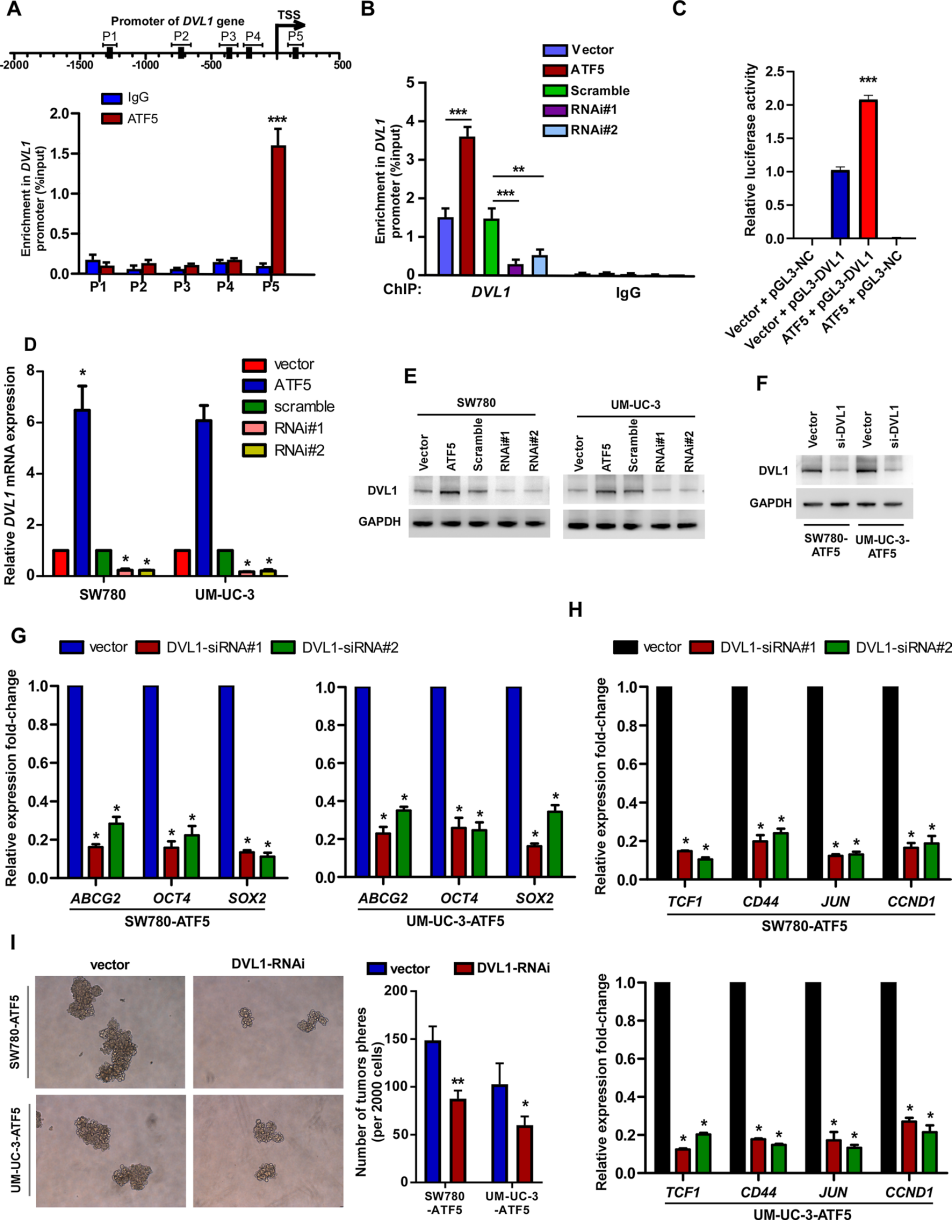
ATF5 directly upregulates *DVL1* in bladder cancer cells. **A** Prediction (upper panel) and validation (lower panel) of potential ATF5-target sites by JASPAR database and ChIP-qPCR assay. **B** ChIP-qPCR analysis showing enrichment of ATF5 at *DVL1* promoter in SW780/UM-UC-3 cells upon ATF5 up-regulation or knock-down. **C** Luciferase reporter test of *DVL1* transcriptional activity upon transfection with ATF5 plasmids and/or luciferase plasmids containing/without *DVL1* promoter. **D**, **E** qRT-PCR (**D**) and WB (**E**) detection of *DVL1* expression in ATF5-upregulation or ATF5-knockdown cells compared with control groups. GAPDH was used as the loading control. **F** WB detection of DVL1 protein in ATF5-overexpressing bladder cancer cells upon transfection with *DVL1* siRNAs. **G** qRT-PCR detection of stemness-related markers (*ABCG2*, *OCT4*, *SOX2*) in ATF5-overepxressing SW780/UM-UC-3 cells upon knockdown of *DVL1*. **H** qRT-PCR assay of genes expression of Wnt/β-catenin downstream targets (*TCF1*, *CD44*, *JUN* and *CCND1*) in ATF5-overepxressing SW780/UM-UC-3 cells transfected with *DVL1* siRNAs. **I** Representative images (left, × 100) and quantitative analyses (right) of tumor sphere formation in ATF5-overexpressig cells upon knockdown of *DVL1*. Bars represent the mean ± SD of three independent experiments; *P < 0.05, **P < 0.01, ***P < 0.001. qRT-PCR, quantitative real-time polymerase chain reaction; WB, western blotting

JASPAR analysis was conducted to identify the potential downstream targets of ATF5 in the Wnt/β-catenin pathway. *DVL1* gene promoters were predicted to include ATF5 binding sites (Fig. 4A). ChIP test validated that ATF5 could directly bind to *DVL1* promoters (Fig. 4A and B). Moreover, ATF5 upregulation enhanced *DVL1* promoter-driven luciferase activity (Fig. 4C) and DVL1 expression (Fig. 4D and E) in bladder cancer cells, while ATF5 knockdown exhibited the opposite effects (Fig. 4C–E). These data further validated that ATF5 could directly bind to and positively stimulate *DVL1*. Subsequently, we explored whether DVL1 expression was necessary for ATF5 to promote the TIC-like phenotype of bladder cancer cells. Knockdown of DVL1 in ATF5-overexpressing cells (Fig. 4F) inhibited the expression level of stemness-related markers, including *OCT4*, *ABCG2*, and *SOX2* (Fig. 4G), and expressions of downstream genes of Wnt/β-catenin pathway (Fig. 4H). Importantly, DVL1 knockdown decreased the sphere-formation capability of ATF5-overexpressing cells (Fig. 4I). These data demonstrated that DVL1 expression was necessary for ATF5 to enhance a TIC-like phenotype of bladder cancer cell.

Altogether, this result showed that ATF5 promoted tumorigenic capability of bladder cancer cells by directly binding and promoting *DVL1* to stimulate the Wnt/β-catenin pathway.

### Clinical correlation of ATF5/DVL1/beta-catenin axis in bladder cancer

**Fig. 5:**
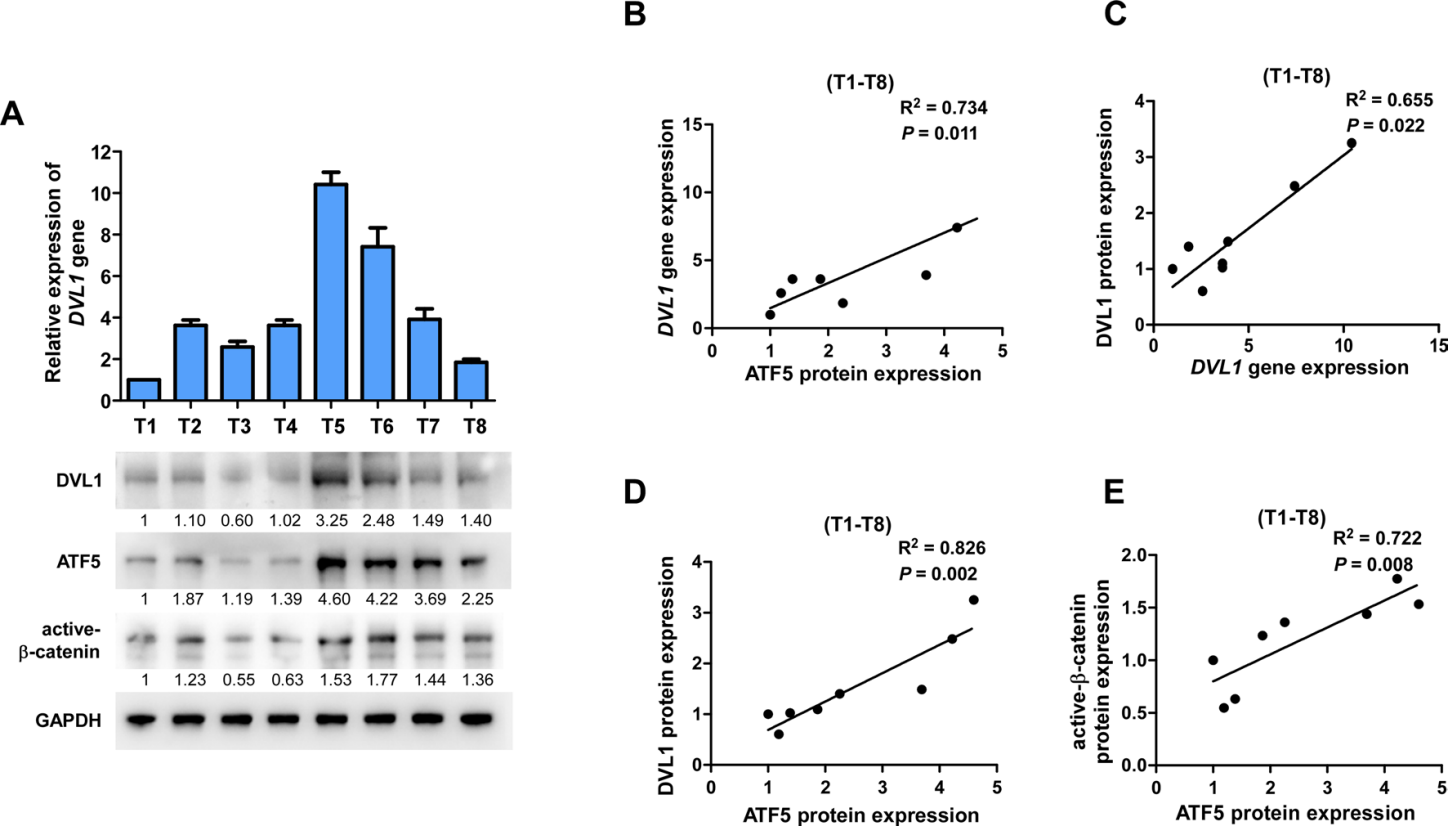
ATF5 expression associated with Wnt/β-catenin signaling stimulation in BLCA. **A** qRT-PCR assay of *DVL1* and WB detection of DVL1, ATF5 and the active-β-catenin in eight fresh BLCA specimens; GAPDH was used as a loading control. **B**–**E** Association analyses of ATF5 expression with expression of DVL1 and the active-β-catenin in eight fresh BLCA specimens. qRT-PCR, quantitative real-time polymerase chain reaction; WB, western blotting; DVL1, dishevelled segment polarity protein 1; BLCA, bladder urothelial carcinoma

Finally, qRT-PCR and WB analyses (Fig. 5A) were performed to check if the Wnt/ATF5/DVL1 axis in bladder cancer cells is of clinical significant. There was a positive association between ATF5 protein and the *DVL1* gene (*P* = 0.011, R^2^ = 0.734), DVL1 protein (*P* = 0.002, R^2^ = 0.826) and the active-β-catenin expression (*P* = 0.008, R^2^ = 0.722) in eight fresh BLCA samples (Fig. 5B–E). In summary, these results showed that ATF5 overexpression in BLCA was positively related to DVL1 expression, which in turn stimulated Wnt/β-catenin pathway to promote tumorigenic capability of bladder cancer cell.

### Aberrant ATF5 amplification is involved in ATF5 overexpression in bladder cancer cells, and is correlated with poor prognostic outcomes

**Fig. 6:**
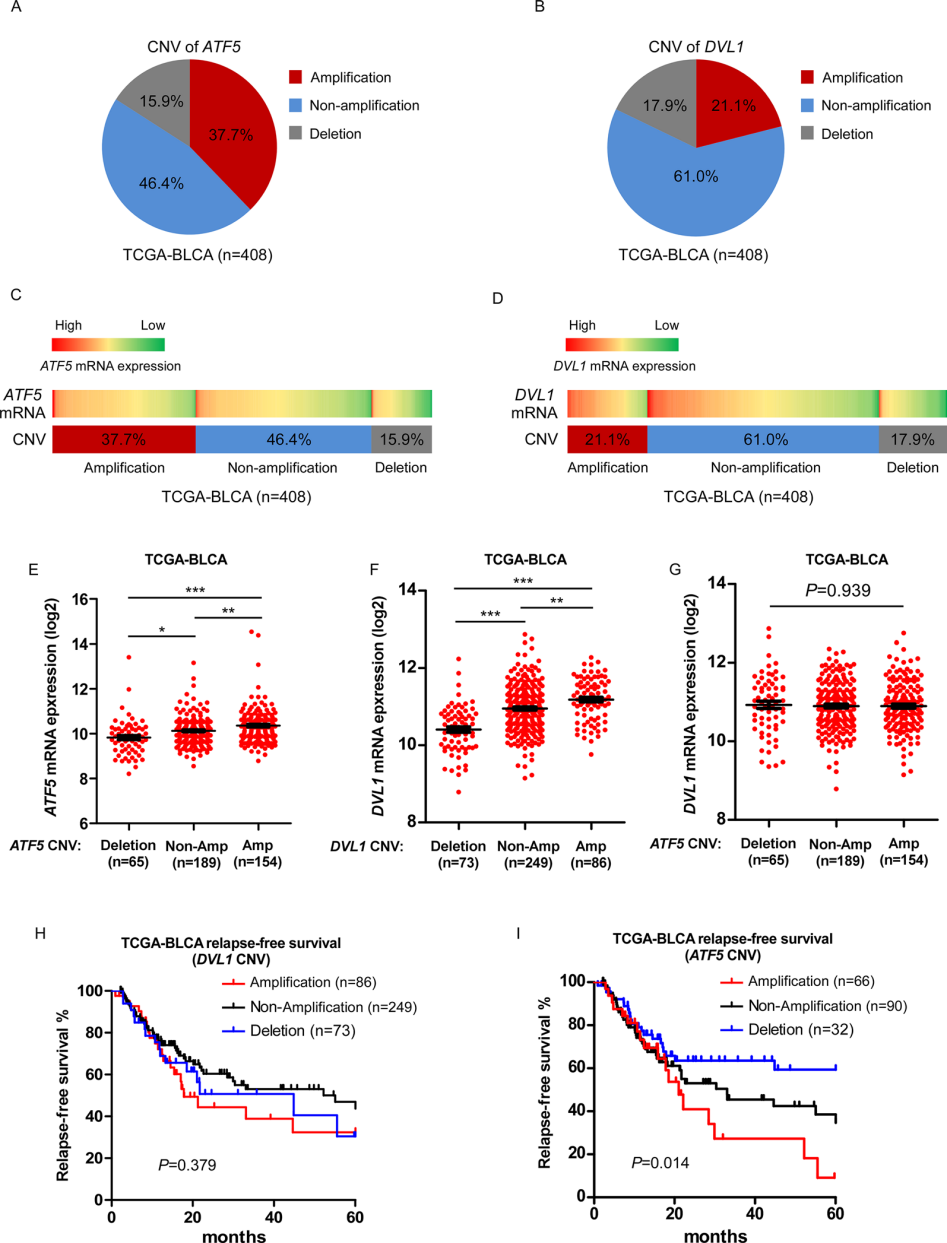
Aberrant *ATF5* amplification contributes to ATF5 overexpression in bladder cancer, which confers poor prognosis. **A**, **B** Analyses of *ATF5* and *DVL1* copy number variant (CNV) in bladder cancer patients from TCGA-BLCA data sets. **C**, **D** *ATF5*, *DVL1* gene CNV and corresponding mRNA expression in bladder cancer patients from TCGA-BLCA data sets. **E**, **F** *ATF5*, *DLV1* gene CNV and corresponding mRNA expression in a TCGA bladder cancer data set (*P* < 0.05). **G** *ATF5* gene CNV and corresponding *DVL1* gene expression in a TCGA bladder cancer data set (*P* = 0.939). **H** Kaplan–Meier analysis of relapse-free survival for patients with amplified, non-amplified or deletion of *DVL1* expression (*P* = 0.379). **I** Kaplan–Meier analysis of relapse-free survival for patients with amplified, non-amplified or deletion of *ATF5* expression (*P* = 0.014). *P < 0.05, **P < 0.01, ***P < 0.001. TCGA, the cancer genome atlas; BLCA, bladder urothelial carcinoma; CNV, copy number variant; Amp, amplification

In various cancers, the *ATF5* locus on chromosome 19q13.33 is generally amplified. The copy number variation (CNV) of ATF5 and DVL1 in TCGA-BLCA datasets were shown in Fig. 6A and B. *ATF5* and *DVL1* mRNA levels were tightly related to the CNV (Fig. 6C and D). The amplification in CNV of *ATF5* and *DVL1* predicted a higher gene expression, while the deletion in CNV of *AFT5* and *DVL1* predicted a lower gene expression (Fig. 6E and F). However, the CNV of *ATF5* was not directly associated with *DVL1* gene expression (Fig. 6G), indicating that *ATF5* CNV does not contribute to the ectopic expression of *DVL1* gene. Although aberrant CNV of *DLV1* contributed to ectopic expression of *DVL1* gene, differences in survival outcomes between *DVL1* amplification or deletion patients were insignificant (*P* = 0.379, Fig. 6H). Notably, bladder cancer with *ATF5* amplification was found to be a predictor of worse survival outcomes than those without *ATF5* amplification (*P* = 0.014, Fig. 6I). These results indicated that aberrant *ATF5* amplification plays a role in ATF5 overexpression in bladder cancer, which conferred a poor prognosis.

## Discussion

This study revealed that ATF5 facilitates the formation of tumor sphere, promotes tumorigenicity and stimulates the Wnt/β-catenin pathway in bladder cancer. Furthermore, ATF5 was upregulated in human BLCA and elevated ATF5 was related to relapse-free survival outcomes, implying that ATF5 might be a potential prognosis marker for BLCA recurrence.

ATF5 has been demonstrated to be highly expressed in undifferentiated neural progenitor/stem cells [16–19]. It seems to repress the differentiation of bone and brain tissues [20, 23], while targeted abrogation of ATF5 leads to normal differentiation in neural progenitor cells [18–20]. These studies support the potential regulation of ATF5 in the differentiation and self-renewal of TICs. In a variety of cancers, ATF5 has also been characterized to be upregulated, such as leukemia, breast cancer and gliomas [25–27]. ATF5, as a transcription factor, functions as an oncogenic role in enhancing cell survival, migration and radioresistance of cancer cells [28, 29]. Herein, ATF5 alteration significantly changed the expression of stemness-associated genes SOX2 and OCT4. SOX2 and OCT4 are transcription factors that control the transcriptional regulatory network in embryonic stem cells [30]. They were commonly used as the markers of CSC in multiple cancers [31, 32], including bladder cancer [33, 34]. Our results indicated that ATF5 up-regulation could promote the stemness of bladder cancer cells. Furthermore, we found that overexpressions of ATF5 in bladder cancer cells promoted the formation of tumor sphere, which was correlated with the relapse-free survival of BLCA patients. On the contrary, the down-regulation of ATF5 inhibited the formation of spheres, which was correlated with the improvement of survival rate. These results indicate that ATF5 overexpression increases tumorigenicity and enhances the TIC-like phenotype in bladder cancer cells, thereby providing hope for developing novel therapeutic strategy to prevent BLCA recurrence.

The Wnt/β-catenin signaling, known as the canonical Wnt pathway, is essential for development of the embryo as well as tissue self-renewal of tissues [35, 36]. Abnormal activations of this pathway can lead to unrestrained cells proliferation and malignant transformation [35, 36]. As one of the most relevant pathways associated with TICs, this pathway is often abnormally stimulated in various cancers, including bladder cancer [13, 37]. Stimulation of Wnt/β-catenin pathway by miR-543-3p could increase [38], whereas inhibition of this signaling by miR-139-5p may inhibit [39] TIC-like phenotype of BLCA cells, supporting the vital roles of Wnt/β-catenin pathway in regulating TIC-like phenotype of bladder cancer. Consistent with these studies, we detected that Wnt/β-catenin signaling was abnormally activated in BLCA. We showed that ATF5 could directly target and positively regulate *DVL1*, leading to the stimulation of Wnt/β-catenin signaling.

DVL1, as a main component of the Wnt pathway, takes part in transduction of Wnt signals to β-catenin, and then stimulates downstream effector factors [40]. In this study, we found that ATF5 could directly bind to *DVL1* promoter and stimulate its expression, and then activate the downstream genes of the Wnt/β-catenin pathway, including active β-catenin, MYC, CD44, JUN as well as CCND1, whereas down-regulating ATF5 reduced the expression of DVL1 and these factors. These findings demonstrate a novel mechanism underpinning hyperactivation of the Wnt/β-catenin pathway in BLCA. Herein, this study indicates that ATF5 could simulate the Wnt/β-catenin pathway and promote tumorigenic capability.

## Conclusion

The present study reveals that overexpression of ATF5 in BLCA directly promotes *DVL1* expression and stimulates the Wnt/β-catenin signaling, therefore increasing tumorigenicity, enhancing a TIC-like phenotype as well as predicting poor survival. Evaluation of the role of ATF5 in BLCA will broaden our understanding of the mechanism underpinning the high recurrence rate of BLCA, and establish whether ATF5 serves as a prognosis marker or potential treatment target for BLCA recurrence.

## Data Availability

The public datasets of BLCA in NCBI (https://www.ncbi.nlm.nih.gov/gene/); gene set enrichment analysis software program (GSEA, http://software.broadinstitute.org/gsea/msigdb/index.jsp).
